# Interoceptive Awareness Among the General Public in Saudi Arabia: A Cross-Sectional Study

**DOI:** 10.7759/cureus.49771

**Published:** 2023-12-01

**Authors:** Ebtihaj Omar Fallata, Kadeja Abdulrahman Bashekah, Reem Mohammed Alqahtani, Sohaib Essam Althagafi, Mohammed Hisham Bardesi, Abdulaziz Mustafa Adnan, Mohammed Ali Alfaqih, Abdulrahman Mauafaq Aljifri, Hind Mauafaq Aljifri

**Affiliations:** 1 Department of Psychiatry, Eradah and Mental Health Complex, Saudi Ministry of Health, Jeddah, SAU; 2 Department of Diabetes and Endocrinology, Endocrine and Diabetes Center, Saudi Ministry of Health, Jeddah, SAU; 3 Department of Family Medicine, Faculty of Medicine, King Abdulaziz University, Jeddah, SAU; 4 Faculty of Medicine, King Abdulaziz University, Jeddah, SAU; 5 Department of Medicine, King Abdulaziz University, Jeddah, SAU; 6 Department of Psychiatry, Faculty of Medicine, King Abdulaziz University, Jeddah, SAU; 7 Faculty of Medicine, Fakeeh College of Medical Sciences, Jeddah, SAU

**Keywords:** survey, saudi arabia, interoception, public, awareness

## Abstract

Background

Interoception refers to the cognitive process of perceiving internal bodily states. This encompasses various physiological indicators, including heart rate fluctuations, stomach distention, internal temperature, hydration levels, sensory input from free nerve terminals in the fascia and muscles, as well as hormonal, stretch, and pain receptors. This study aimed to examine the interoceptive awareness among the general public in Saudi Arabia.

Methods

A cross-sectional online survey was undertaken in Saudi Arabia to investigate the level of interoceptive awareness within the overall population of the country in October 2023. This research used a previously developed questionnaire named the Multidimensional Assessment of Interoceptive Awareness, version 2 (MAIA-2). In a binary logistic regression analysis, the mean interoceptive awareness score of the participants was utilized as the dummy variable to determine the variables that influence interoceptive awareness.

Results

A total of 814 participants were involved in this study. Overall, the study participants demonstrated a marginal level of interoceptive awareness with a mean score of 94.3 (standard deviation (SD): 29.3) out of 185 (representing 51.0% of the maximum attainable score). The mean interoceptive score was not consistent across different subscales and ranged between 37.7% and 63.3%. The highest mean interoceptive score was observed for the Trusting subscale (9.5 (SD: 4.5) out of 15) (representing 63.3% of the maximum attainable score for this subscale). The lowest mean interoceptive score was observed for the Not-Distracting subscale (11.3 (SD: 6.9) out of 30) (representing 37.7% of the maximum attainable score for this subscale). Binary logistic regression analysis did not identify any statistically significant difference in the likelihood of having a higher level of interoceptive awareness among the participants based on their demographic characteristics (p>0.05).

Conclusion

The participants in our research demonstrated a modest degree of interoceptive awareness. The study's results suggest that the participants demonstrated a heightened inclination towards internal experiences rather than being attentive to their bodily sensations. Further investigation is required to examine interoceptive awareness across various cohorts.

## Introduction

Interoception pertains to the perception of interior bodily states, encompassing several physiological indicators such as fluctuations in heart rate, stomach distention, internal temperature, hydration levels, sensory input from free nerve terminals in the fascia and muscles, as well as hormonal, stretch, and pain receptors [1]. The concept of interoception is closely linked to emotion and motivation, since it pertains directly to the body's homeostatic condition. It plays a crucial role in our perception of self, consciousness, and overall well-being, including mental well-being [2-6].

Affective and motivational states might be conceptualized as emerging from the interpretations of and alterations in interoceptive signals [6, 7]. For example, certain physiological states such as dehydration or the accumulation of carbon dioxide in the bloodstream can elicit sensations of fear via the process of interoception [8]. Sufficient interoceptive awareness (IA), sensitivity, and precision play a crucial role in self-regulation, enabling the brain to generate homeostatic predictions regarding present and future requirements and subsequently take appropriate measures to fulfill those needs (such as resting or consuming fluids) [6, 9]. Consequently, a diminished capacity for interoceptive sensitivity or awareness might result in increased ambiguity and difficulty in regulating internal states. Nevertheless, an excessive amount of interoceptive sensitivity or awareness can also have negative consequences. This is because heightened interoception can result in overpowering or invasive feelings that offer little adaptive benefit [10]. Therefore, there is a spectrum of interoceptive capacity that includes persons who exhibit hypo-awareness or sensitivity as well as those who exhibit hyper-awareness or sensitivity.

Consistent with the significance of interoception in facilitating adaptive self-regulation [9, 10], contemporary theories of psychopathology propose that an individual's inability to obtain accurate, consistent, or dependable information regarding their internal state (i.e., impaired or disrupted interoception and deficient integration of bodily and neural processes) or distortions in the interpretation of these interoceptive signals can result in substantial challenges in adaptive regulation, such as anxiety and depression [3, 6, 9]. Disorders such as anxiety or depression, sleep disorders, obsessive-compulsive disorder, eating disorders, addiction, certain physical conditions, and challenges with social interactions can be comprehended within this framework as disruptions in the capacity to process and incorporate interoceptive information, resulting in a limited foundation for adaptive predictions and essential self-regulation [4, 11-16]. Consistent with this viewpoint, the majority of mental disorders exhibit diverse issues related to autonomic dysfunctions and emotion dysregulation [17-19]. Additionally, disrupted interoception is linked to several mental diseases and is also associated with a higher likelihood of developing psychopathology in the future [3, 20, 21]. Moreover, recent studies indicate that therapies, such as interoceptive training, have the potential to mitigate symptoms of anxiety and depression while also enhancing overall functioning [22-24].

The investigation of several facets of interoception, encompassing interoceptive consciousness, holds significance in comprehending mental well-being. Psychology of the general public was the interest of previous research in multiple studies in the Middle East region [25-31]. However, there are limited studies that examined this area of research worldwide and in the Middle East specifically. Therefore, this research aims to examine the interoceptive awareness among the general public in Saudi Arabia. The specific objectives are: (1) To assess the level of interoceptive awareness among the general public in Saudi Arabia. (2) To identify interoceptive awareness predictors among the general public in Saudi Arabia.

## Materials and methods

Study design and settings

A cross-sectional online survey was undertaken in Saudi Arabia to investigate the level of interoceptive awareness within the overall population of the country in October 2023.

Sampling procedure

The sample for this research was selected through a method referred to as convenience sampling. This type of sampling falls within the category of non-probability sampling. The present study encompassed individuals who satisfied the predetermined criteria for inclusion and expressed their willingness to partake in the research. At the commencement of the questionnaire, participants were presented with an informed consent form, affording them the opportunity to either proceed with their involvement in the study or opt to withdraw from it. In order to enhance patients' understanding of the significance of their involvement, the research objectives were clearly presented in their entirety. The invitation letter for the study provided a detailed overview of the inclusion criteria.

Study population and recruitment

The population for this study comprised individuals who are at least 18 years old and are residents of Saudi Arabia, belonging to the general community. There are no restrictions based on the gender. Any participant who did not meet the inclusion criteria or did not provide consent for participation was excluded from the study. The survey hyperlink was disseminated across several social media platforms, including Facebook, Snapchat, WhatsApp, and Twitter, with the aim of fostering increased engagement and involvement.

Study tool

This research used a previously developed questionnaire named the Multidimensional Assessment of Interoceptive Awareness, version 2 (MAIA-2) to examine the interoceptive awareness among the general public in Saudi Arabia [32, 33]. The MAIA-2 is a questionnaire consisting of 37 items that are categorized into eight distinct subscales (See the Appendices). The constructs of the study include: (1) Noticing (four items), which refers to the ability to recognize and be aware of neutral, pleasant, and uncomfortable bodily sensations; (2) Not-Distracting (six items), which pertains to the tendency to either ignore or acknowledge bodily sensations of discomfort or pain; (3) Not-Worrying (five items), which involves the capacity to maintain emotional balance despite experiencing sensations of discomfort or pain; (4) Emotional Awareness (five items), which relates to the awareness of the connection between bodily sensations and emotional states; (5) Attention Regulation (seven items), which encompasses the capability to control or sustain attention towards bodily sensations; (6) Body Listening (three items), which refers to the ability to actively listen to one's body sensations for insight; (7) Self-Regulation (four items), which involves the ability to alleviate distress by directing attention towards bodily sensations; and (8) Trusting (three items), which pertains to the experience of perceiving one's body sensations as reliable and safe sources of information [33]. The MAIA-2 questionnaire comprises a total of 37 items, each of which is responded to using a 6-point Likert scale. The scale ranges from 0, representing "never," to 5, representing "always." It is worth noting that nine of the items are reverse-scored. The initial iteration of MAIA-2 exhibited Cronbach's alpha coefficients ranging from 0.64 to 0.83 across its eight scales [33]. Two variables, namely Noticing (0.64) and Not Worrying (0.67), fell below the established threshold of 0.70. All correlations between items and the scale met the predetermined criteria of 0.30 [33].

The items in the study were evaluated using a Likert scale consisting of six points. The scale ranged from 0, indicating "never," to 5, indicating "always." Items 5, 6, 7, 8, 9 and 10 on Not-Distracting, and items 11, 12 and 15 on Not-Worrying are reverse-scored. Higher scores on the scale were indicative of increased levels of self-reported interoceptive awareness, which was considered to be more advantageous [32, 33]. In addition, participants’ demographic characteristics (age, gender, nationality, education, monthly income category, smoking status, employment status, and marital status) were collected from the study participants.

Survey translation

To promote the involvement of the general population in Saudi Arabia, the Arabic version of the questionnaire instrument was used [32]. The validity of the original 8-factor structure of the MAIA-2 was supported by confirmatory factor analyses conducted on the Arabic version. The internal consistency of the Arabic version was assessed and found to be reliable, as indicated by McDonald's ω coefficients ranging from 0.86 to 0.93 for the subscales. The present study examines the measurement invariance of the Arabic version of the MAIA-2 across gender. In conclusion, the dimensions of the Arabic MAIA-2 exhibited significant positive correlations with the intuitive eating component known as "Reliance on Hunger and Satiety Cues," hence offering empirical evidence in favor of convergent validity.

Piloting of the questionnaire tool

The questionnaire instrument was evaluated and validated by medical professionals affiliated with the Saudi Ministry of Health. The participants were queried regarding the clarity, comprehensibility, and face validity of the questions, in addition to any difficulties encountered in understanding them. Furthermore, the participants were requested to provide feedback regarding any inquiries that they perceived as unpleasant. Furthermore, a preliminary investigation was conducted using a limited sample from the target audience to assess their comprehension of the survey instrument prior to its extensive implementation.

Sample size

The minimum required sample size was 385 individuals using a 95% confidence interval, a 0.5 standard deviation (SD), and a 5% margin of error.

Ethical approval

This study was reviewed and approved by the Institutional Review Board at the Ministry of Health, Jeddah, Saudi Arabia (Ref: A01744).

Statistical analysis

Using SPSS version 27 (IBM Corp., Armonk, NY, USA), this study's data were analysed. A histogram and normality metrics were used to examine the normality of interoceptive awareness score. Based on the normality of the data, interoceptive awareness score was presented as the mean (SD). In a binary logistic regression analysis, the mean interoceptive awareness score of the participants (which was equal to 94.3 (standard deviation: 29.3)) was utilized as the dummy variable to determine the variables that influence interoceptive awareness. To determine statistical significance, a two-sided p-value less than 0.05 was utilized.

## Results

A total of 814 participants were involved in this study. More than half of the participants (56.4%; n=459) were females and married (62.9%; n=512). Around one-quarter (23.1%; n=188) of the participants were aged 41-50 years. Around 56.6% (n=461) of the participants reported that they hold a diploma. Around one-quarter (24.3%; n=198) of the participants reported that their family monthly income category is between 5001 and 10000 Saudi Arabia riyal (SAR). Almost one-third (34.2%; n=278) of the study participants reported that they work outside the healthcare sector. The vast majority of the study participants (92.3%; n=751) were Saudis. Almost one-fifth (22.7%; n=185) of the study participants reported that they are current smokers. Besides, around one-third (30.3%; n=247) of the study participants reported that they have other comorbidity history. For further details on the demographic characteristics of the study participants, refer to Table 1.

**Table 1:** Participants' demographic characteristics. SAR: Saudi Arabia riyal; The data has been represented as frequency and percentage.

Variable	Frequency	Percentage
Gender		
Females	459	56.4%
Age categories		
18-23 years	78	9.6%
24-30 years	151	18.6%
31-40 years	143	17.6%
41-50 years	188	23.1%
51-60 years	177	21.7%
61 years and older	77	9.5%
Marital status		
Single	239	29.4%
Married	512	62.9%
Divorced	45	5.5%
Widowed	18	2.2%
Education		
Secondary school or lower	110	13.5%
Diploma	461	56.6%
Bachelor’s degree	164	20.1%
Higher education	79	9.7%
Family monthly income		
Less than 5000 SAR	138	17.0%
5001-10000 SAR	198	24.3%
10001-15000 SAR	164	20.1%
15001-20000 SAR	151	18.6%
20001 SAR and above	163	20.0%
Employment status		
Retired	141	17.3%
Unemployed	180	22.1%
Working in the healthcare sector	164	20.1%
University student	51	6.3%
Working outside the healthcare sector	278	34.2%
Nationality		
Saudi	751	92.3%
Current smokers (Yes)	185	22.7%
Comorbidity history (Yes)	247	30.3%

Interoceptive awareness profile

Table 2 presents the mean interoceptive awareness score stratified by sub-scale. Overall, the study participants demonstrated a marginal level of interoceptive awareness with a mean score of 94.3 (SD: 29.3) out of 185 (representing 51.0% of the maximum attainable score). The mean interoceptive score was not consistent across different subscales and ranged between 37.7% and 63.3%. The highest mean interoceptive score was observed for the Trusting subscale (9.5 (SD: 4.5) out of 15) (representing 63.3% of the maximum attainable score for this subscale). The lowest mean interoceptive score was observed for Not-Distracting subscale (11.3 (SD: 6.9) out of 30) (representing 37.7% of the maximum attainable score for this subscale).

**Table 2:** Mean interoceptive awareness score stratified by sub-scale The data has been represented as Mean ± Standard deviation and as a percentage of the total score for each sub-scale.

Scale	Questions	Mean score (standard deviation)	Percentage of maximum attainable score
Noticing score	Q1-Q4	10.7 (5.5)	53.5%
Not-Distracting score	Q5-Q10	11.3 (6.9)	37.7%
Not-Worrying score	Q11-Q15	10.8 (4.2)	43.2%
Attention Regulation score	Q16-Q22	18.0 (8.4)	51.4%
Emotional Awareness score	Q23-Q27	15.5 (6.9)	62.0%
Self-Regulation score	Q28-Q31	11.0 (5.4)	55.0%
Body Listening score	Q32-Q34	7.5 (4.3)	50.0%
Trusting score	Q35-Q37	9.5 (4.5)	63.3%
Overall interoceptive awareness score	Q1-Q37	94.3 (29.3)	51.0%

Predictors of a higher level of interoceptive awareness

Binary logistic regression analysis did not identify any statistically significant difference in the likelihood of having a higher level of interoceptive awareness among the participants based on their demographic characteristics (p>0.05) (Table 3).

**Table 3:** Predictors of higher level of interoceptive awareness SAR: Saudi Arabia riyal; The data has been represented as odds ratio with 95% confidence interval; *p<0.05, **p<0.01; ***p<0.001

Variable	Odds ratio of having a higher level of interoceptive awareness	P-value
Gender		
Females (Reference group)	1.00	
Males	0.78 (0.59-1.03)	0.077
Age categories		
18-23 years (Reference group)	1.00	
24-30 years	1.06 (0.61-1.84)	0.850
31-40 years	1.08 (0.62-1.90)	0.787
41-50 years	0.96 (0.56-1.64)	0.881
51-60 years	0.97 (0.56-1.66)	0.907
61 years and older	0.93 (0.49-1.76)	0.817
Marital status		
Single (Reference group)	1.00	
Married	0.92 (0.67-1.26)	0.590
Divorced	0.68 (0.36-1.29)	0.233
Widowed	1.30 (0.47-3.57)	0.616
Education		
Secondary school or lower (Reference group)	1.00	
Bachelor’s degree	1.00 (0.65-1.52)	0.986
Higher education	1.14 (0.70-1.87)	0.606
Diploma	0.75 (0.42-1.34)	0.326
Family monthly income		
Less than 5000 SAR (Reference group)	1.00	
5001-10000 SAR	1.12 (0.72-1.74)	0.628
10001-15000 SAR	0.97 (0.62-1.54)	0.909
15001-20000 SAR	0.93 (0.59-1.49)	0.773
20001 SAR and above	1.18 (0.74-1.88)	0.481
Employment status		
Retired (Reference group)	1.00	
Unemployed	0.95 (0.61-1.49)	0.823
Working in the healthcare sector	1.06 (0.67-1.68)	0.803
University student	0.60 (0.32-1.15)	0.124
Working outside the healthcare sector	1.02 (0.68-1.54)	0.922
Nationality		
Saudi (Reference group)	1.00	
Non-Saudi	1.55 (0.89-2.68)	0.121
Current smoker		
No (Reference group)	1.00	
Yes	1.30 (0.93-1.82)	0.130
Comorbidity history		
No (Reference group)	1.00	
Yes	0.79 (0.59-1.07)	0.135

## Discussion

Interoception is the mechanism through which the nervous system detects, understands, and combines signals arising from within the body. This process creates a continuous representation of the body's internal state, operating both consciously and unconsciously [3]. Indeed, interoception involves sensing several physiological indicators in our internal body states like heart rate, stomach fullness, and temperature, and connects to emotions and motivation, influencing how we perceive ourselves and our well-being [1, 6]. Therefore, this study aimed to examine the interoceptive awareness among the general public in Saudi Arabia.

This study was conducted using MAIA-2 tool to assess the level of interoceptive awareness among the study participants, where MAIA tool holds significant advantages that allow more differentiated assessment of essential psychological aspects of the perception and evaluation of body sensations [3]. Furthermore, MAIA is considered as one of the few tools that enable a comprehensive assessment of interoceptive body awareness by involving multiple dimensions of analysis [34]. Indeed, the MAIA comprises eight scales that encompass five key dimensions of body awareness, where these scales provide a comprehensive understanding of different aspects of body awareness through eight subscales including “Noticing”, which involves being aware of body sensations, and “Not-Distracting” and “Not-Worrying”, which relate to emotional reactions and attentional responses to sensations. “Attention Regulation” measures the capacity to regulate attention, while “Emotional Awareness” focuses on understanding mind-body integration. Additionally, “Self-Regulation” and “Body Listening” further explore the awareness of mind-body integration. Lastly, the “Trust” scale evaluates one's confidence in trusting body sensations [33].

Most of the previous research in Iran [35], Korea [36], Japan [37], Colombia [34], and Turkey [38] examined the MAIA reliability and validation to be used and applied among different study populations. This tool was validated to suit populations with different languages, and also to suit different sociocultural groups, where these validations allow clinicians and researchers to use MAIA among these populations [34-39].

In this study, the participants exhibited a marginal level of interoceptive awareness, with an overall mean score of 94.3 out of 185, indicating that they achieved approximately 51.0% of the maximum attainable score. In fact, the level of self-awareness may vary from culture to culture, depending on how individuals process their own interoceptive self-information [40], where bodily sensation and interoceptive awareness level are found to be mediated and controlled by the right anterior insular cortex that contributes the intensity of negative emotions [41]. However, after examining the relationship between interoceptive awareness and emotional susceptibility, it was found that there is an independence between interoceptive accuracy and interoceptive awareness measures [42]. Indeed, the variation in the awareness scores across different subscales highlights the heterogeneity in individuals' awareness of their internal bodily sensations, where lack of interoceptive awareness and low interoceptive sensitivity were found to be associated with self-objectification [43].

After examining the scores on different subscales, this study found a nuanced understanding of interoceptive awareness, where the “Trusting” subscale, where individuals trust their internal sensations, showed the highest mean score (63.3% of the maximum attainable score). Therefore, self-trust is found to be important to understand how individuals may interpret and respond to any interoceptive stimuli [44], where self-trust is a crucial prerequisite for both personal independence and self-esteem [45], and it shows how individuals reflect and interpret their emotional response [46].

On the other hand, the “Not-Distracting” subscale, indicating the ability to not distract oneself from uncomfortable sensations, exhibited the lowest mean score (37.7% of the maximum attainable score), where this suggests that while individuals might be attuned to their bodily sensations, they struggle with managing or accepting certain discomforts, leading to distractions, in fact, avoiding or distracting attention from emotion-related physical sensations seems logical when someone refuses to acknowledge negative feelings, where the connection with trait anxiety suggests that individuals with this trait might tend to overlook discomforting bodily sensations [47].

This study underscores the importance of cultivating self-trust and managing emotional discomforts, offering valuable insights into the interplay of internal sensations and mental well-being within the Saudi Arabian context. The concept of self-awareness is frequently linked to enhanced emotional control and overall psychological well-being [48]. Individuals who exhibit lower susceptibility to distraction may potentially enhance their mental well-being by cultivating mindfulness towards their emotions and body sensations [48]. The presence of heightened concern for body sensations can potentially contribute to the development and exacerbation of anxiety and stress. Individuals exhibiting lower levels of worry may demonstrate greater resilience in mitigating the adverse effects of interior sensations on their overall mental well-being [48]. Individuals who possess the ability to redirect and regulate their attention away from unpleasant bodily sensations may exhibit more proficiency in stress management and the preservation of mental well-being [48]. The practice of body listening has the potential to augment self-awareness and yield significant insights on an individual's mental and physical condition. The increased level of consciousness facilitates enhanced emotional regulation, hence leading to enhanced mental well-being. Individuals who possess the ability to proficiently regulate their emotional and physiological reactions are more adept at managing challenges and preserving their psychological well-being [48].

Future research could delve deeper into the cultural and social factors influencing interoceptive awareness, where longitudinal studies might track changes in interoceptive awareness over time or following specific interventions, providing valuable insights into the malleability of this construct. Moreover, qualitative research methods, such as interviews or focus groups, may offer a richer understanding of individuals' experiences related to interoceptive awareness, as well as future interventions could focus on enhancing self-trust and coping mechanisms to promote holistic self-awareness and emotional resilience among individuals in this population.

The main limitation of this study is the cross-sectional study design, which restricted our ability to examine causality among the study variables. In addition, there are limited studies that examined this research area, which restricted our ability to make comparison with other study populations.

## Conclusions

The participants in our study exhibited a marginal level of interoceptive awareness. The findings of the study indicate that the individuals involved exhibited a greater reliance on their internal sensations as opposed to being attentive to their body sensations. The results of our investigation revealed a lack of appropriate interoceptive awareness. Furthermore, the results of the study highlight the significance of including therapies that especially focus on interoceptive awareness within clinical settings. Additional research is necessary to investigate interoceptive awareness among different cohorts. Gaining insight into the progression of interoceptive awareness during different stages of life, as well as its modulation in relation to significant life events or therapeutic interventions, might provide valuable knowledge for the design and implementation of mental health therapies that are more precise and efficacious. Further research should investigate the impact of cultural norms, social situations, and environmental elements on individuals' subjective experiences of interior sensations. This can provide valuable insights for the development of culturally sensitive therapies and lead to a more comprehensive and nuanced comprehension of interoceptive awareness.

## Appendices

**Table 4:** Multidimensional Assessment of Interoceptive Awareness Version 2

Below you will find a list of statements. Please indicate how often each statement applies to you generally in daily life.	Circle one number on each line						
1. When I am tense I notice where the tension is located in my body.		0	1	2	3	4	5
2. I notice when I am uncomfortable in my body.		0	1	2	3	4	5
3. I notice where in my body I am comfortable.		0	1	2	3	4	5
4. I notice changes in my breathing, such as whether it slows down or speeds up.		0	1	2	3	4	5
5. I ignore physical tension or discomfort until they become more severe.		0	1	2	3	4	5
6. I distract myself from sensations of discomfort.		0	1	2	3	4	5
7. When I feel pain or discomfort, I try to power through it.		0	1	2	3	4	5
8. I try to ignore pain		0	1	2	3	4	5
9. I push feelings of discomfort away by focusing on something		0	1	2	3	4	5
10. When I feel unpleasant body sensations, I occupy myself with something else so I don’t have to feel them.		0	1	2	3	4	5
11. When I feel physical pain, I become upset.		0	1	2	3	4	5
12. I start to worry that something is wrong if I feel any discomfort.		0	1	2	3	4	5
13. I can notice an unpleasant body sensation without worrying about it.		0	1	2	3	4	5
14. I can stay calm and not worry when I have feelings of discomfort or pain.		0	1	2	3	4	5
15. When I am in discomfort or pain I can’t get it out of my mind		0	1	2	3	4	5
16. I can pay attention to my breath without being distracted by things happening around me.		0	1	2	3	4	5
17. I can maintain awareness of my inner bodily sensations even when there is a lot going on around me.		0	1	2	3	4	5
18. When I am in conversation with someone, I can pay attention to my posture.		0	1	2	3	4	5
19. I can return awareness to my body if I am distracted.		0	1	2	3	4	5
20. I can refocus my attention from thinking to sensing my body.		0	1	2	3	4	5
21. I can maintain awareness of my whole body even when a part of me is in pain or discomfort.		0	1	2	3	4	5
22. I am able to consciously focus on my body as a whole.		0	1	2	3	4	5
23. I notice how my body changes when I am angry.		0	1	2	3	4	5
24. When something is wrong in my life I can feel it in my body.		0	1	2	3	4	5
25. I notice that my body feels different after a peaceful experience.		0	1	2	3	4	5
26. I notice that my breathing becomes free and easy when I feel comfortable.		0	1	2	3	4	5
27. I notice how my body changes when I feel happy/joyful.		0	1	2	3	4	5
28. When I feel overwhelmed I can find a calm place inside.		0	1	2	3	4	5
29. When I bring awareness to my body I feel a sense of calm.		0	1	2	3	4	5
30. I can use my breath to reduce tension.		0	1	2	3	4	5
31. When I am caught up in thoughts, I can calm my mind by focusing on my body/breathing.		0	1	2	3	4	5
32. I listen for information from my body about my emotional state.		0	1	2	3	4	5
33. When I am upset, I take time to explore how my body feels.		0	1	2	3	4	5
34. I listen to my body to inform me about what to do.		0	1	2	3	4	5
35. I am at home in my body.		0	1	2	3	4	5
36. I feel my body is a safe place.		0	1	2	3	4	5
37. I trust my body sensations.		0	1	2	3	4	5
